# Autism-Specific Covariation in Perceptual Performances: “*g*” or “*p*” Factor?

**DOI:** 10.1371/journal.pone.0103781

**Published:** 2014-08-12

**Authors:** Andrée-Anne S. Meilleur, Claude Berthiaume, Armando Bertone, Laurent Mottron

**Affiliations:** 1 The University of Montreal Center of Excellence for Pervasive Developmental Disorders (CETEDUM), Hôpital Rivière-des-Prairies, Montreal, Quebec, Canada; 2 School/Applied Child Psychology, Department of Education and Counselling Psychology, McGill University, Montreal, Quebec, Canada; University of Verona, Italy

## Abstract

**Background:**

Autistic perception is characterized by atypical and sometimes exceptional performance in several low- (e.g., discrimination) and mid-level (e.g., pattern matching) tasks in both visual and auditory domains. A factor that specifically affects perceptive abilities in autistic individuals should manifest as an autism-specific association between perceptual tasks. The first purpose of this study was to explore how perceptual performances are associated within or across processing levels and/or modalities. The second purpose was to determine if general intelligence, the major factor that accounts for covariation in task performances in non-autistic individuals, equally controls perceptual abilities in autistic individuals.

**Methods:**

We asked 46 autistic individuals and 46 typically developing controls to perform four tasks measuring low- or mid-level visual or auditory processing. Intelligence was measured with the Wechsler's Intelligence Scale (FSIQ) and Raven Progressive Matrices (RPM). We conducted linear regression models to compare task performances between groups and patterns of covariation between tasks. The addition of either Wechsler's FSIQ or RPM in the regression models controlled for the effects of intelligence.

**Results:**

In typically developing individuals, most perceptual tasks were associated with intelligence measured either by RPM or Wechsler FSIQ. The residual covariation between unimodal tasks, i.e. covariation not explained by intelligence, could be explained by a modality-specific factor. In the autistic group, residual covariation revealed the presence of a plurimodal factor specific to autism.

**Conclusions:**

Autistic individuals show exceptional performance in some perceptual tasks. Here, we demonstrate the existence of specific, plurimodal covariation that does not dependent on general intelligence (or “g” factor). Instead, this residual covariation is accounted for by a common perceptual process (or “p” factor), which may drive perceptual abilities differently in autistic and non-autistic individuals.

## Introduction

In addition to socio-communicative alterations, autistic individuals present lifelong behavioural characteristics related to visual and auditory perception [1]. These include hypersensitivity to noise [2], prolonged visual exploration of objects [3], early preference for geometric figures over social information [4], and early detection of cross-modal synchrony [5]. The prominence of these behaviours has led to the inclusion of sensory atypicalities and behaviours among the diagnostic criteria for the Autism Spectrum Disorder in the latest version of the Diagnostic and Statistical Manual of Mental Disorders (DSM-5) [6]. Note that, in keeping with the current consensus on language in autism research, the term “autistic” rather than “person with autism” is employed in a respectful way [7] [8].

Atypical perceptual abilities have also been demonstrated in experimental settings with tasks that assess low- and mid-level information processing. Low-level refers to the early stages of information processing upon entry into the perceptual system. This is mediated by primary cortical areas (e.g., visual area V1) that extract elementary perceptual dimensions (e.g., luminance-contrast, spatial frequency, pitch.) and send feedforward signals to mid-level cortical systems for further processing. Autistic atypicalities are mostly characterized by exceptionable extraction of low-level physical dimensions of auditory [9]–[12] (see Bhatara et al. 2013 [13]) and visual [14]–[18] (see Schwarzkopf et al. 2014 [19]) information. As a result, autistic individuals perform better in discrimination tasks than age- and intelligence-matched typically developing participants.

Mid-level perceptual mechanisms involve later stages of perceptual processing (i.e., extra striate, associative cortical areas, etc.) and the integration of low-level signals and grouping processes (e.g., pattern recognition and manipulation). Mid-level information processing is more susceptible than low-level systems to the influences of expectations and semantic knowledge. In autism, high performance in mid-level tasks is primarily the result of a non-mandatory influence of global/gestalt effects. High performances are consistently demonstrated during visuospatial tasks requiring pattern extraction, detection, matching and/or manipulation [20], [21], but have also been documented in the auditory domain during musical tasks [22]–[24]. Some of these perceptual capabilities are evident as early as three years of age [25], indicating that high perception in autistic individuals manifests at various steps of processing in different modalities and relatively early in development.

Knowledge of how perceptual performances are associated within or across levels of processing and/or modalities is crucial to determine whether altered autistic perception results from the effect of a factor, or atypical process, specific to autism. Although high perceptual processing in both auditory and visual modalities has been associated with autism, most studies demonstrating autistic perceptual alterations have examined one modality or level in isolation. Therefore, it remains unknown whether high processing in a particular domain of perception is related to performance in other perceptual functions, levels and modalities, despite the frequent assumption that this is the case [26], [27].

There is a correlation between perceptual and other cognitive abilities in typically developing individuals. Spearman used factorial analyses to suggest that correlation between diverse cognitive abilities may be explained by a general intelligence factor, which he labelled the “g-factor” [28]. In autism, recent data suggest that perception makes a strong contribution to intelligence and that its high autonomy involves the optional use of higher cortical areas (e.g., low functional activation of prefrontal areas) during the processing of perceptual and non-perceptual information [29]. The identification of a relationship between perceptual abilities, besides general intelligence in autistic individuals but not in typically developing individuals would provide evidence for a perceptual factor specific to autism. The existence of a general perceptual factor would be consistent with the Enhanced Perceptual Functioning (EPF) model of autistic cognition [30], [31]. According to the EPF model, autistic cognition is characterized by a bottom-up processing style dominated by the strong activation of early neural mechanisms across perceptual modalities [32] and autonomy of perceptual processes toward top-down influences (i.e., a weak effect of expectations on percepts such as visual illusions [33]).

Research on autistic perceptual strengths and weaknesses has been largely conducted with control groups most frequently matched to the autism group with the Wechsler scale (i.e, FSIQ), and, to a lesser extent, Raven's Progressive Matrices (RPM) as a measure of intelligence [34]. The RPM assesses fluid intelligence, which is strongly associated with general intelligence in typical development. It is administered as a series of multiple-choice questions, requiring no verbal instructions. In contrast, the Wechsler Intelligence Scale, which is based on a multidimensional theory of intelligence, assesses different cognitive abilities to measure overall intellectual performance. Several of its subtests assess comprehension and verbal expression skills. Although the Wechsler Intelligence Scale is the most commonly used tool for cognitive assessment, RPM may be more suitable to measure intelligence in some people with a handicap that alters the encoding of information. For instance, children with a hearing impairment perform in the average range on the RPM, whereas they perform in the range for intellectual disability on the Wechsler verbal IQ scale [35]. This result supports the importance of using a measure of general intelligence tailored to the population being tested to obtain an adequate estimate of overall intellectual capacity without bias from secondary factors.

Although matching autistic and non-autistic groups with an intelligence measure is necessary to control for general cognitive ability between groups, the method used to match intelligence may also significantly affect the results and their interpretation. In some cases, significant differences in the performance of perceptual tasks between groups matched on Wechsler Full Scale IQ disappear when the same groups are matched on Raven Progressive Matrices [36], a measure that is considered by some as a more accurate reflection of autistic intelligence [37], [38]. This lack of equivalence between measures of intelligence that are strongly correlated in typical development further suggests that the components of general intelligence in autism may differ from those in non-autistic individuals.

**Figure 1:**
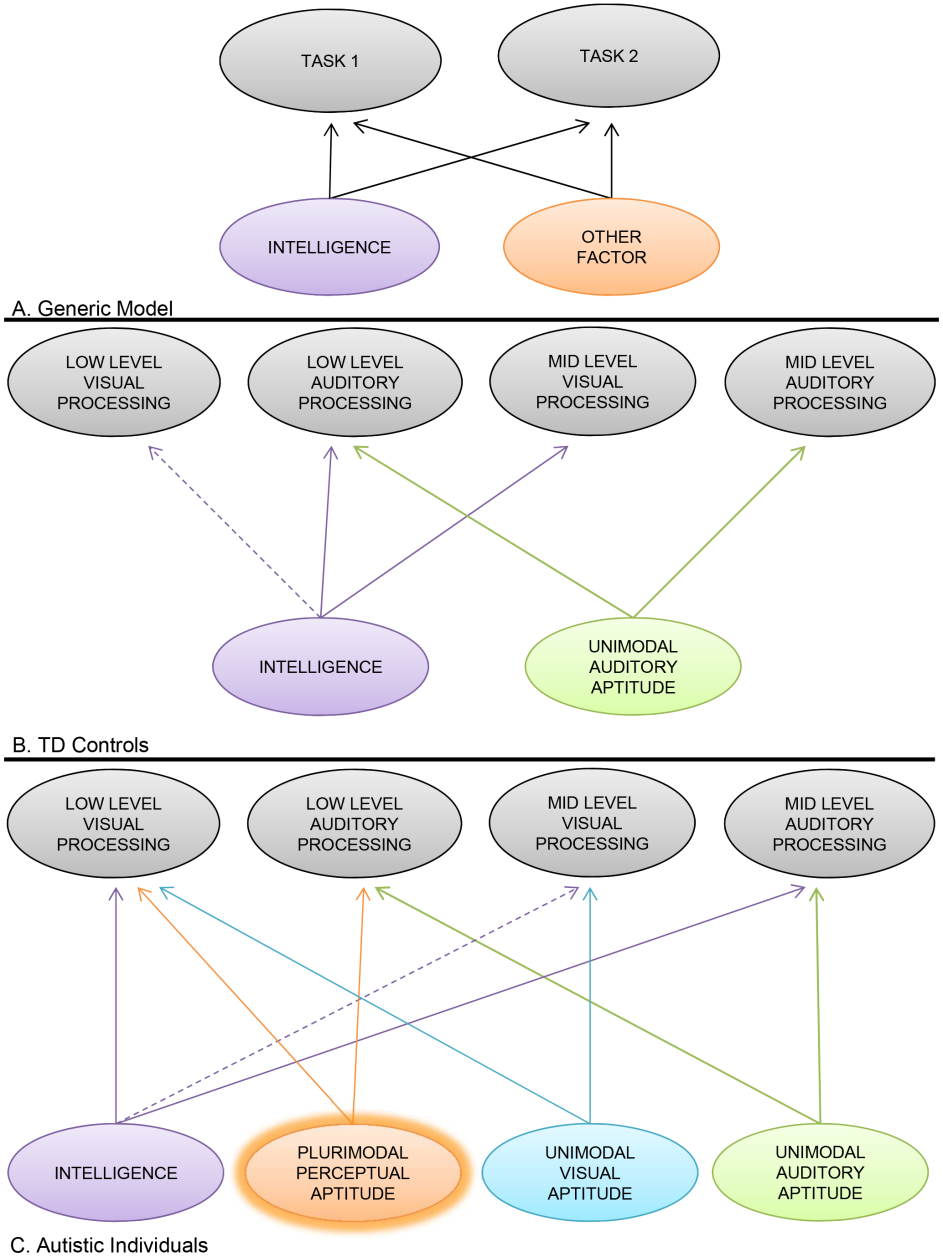
Illustration of theoretical models to explain the pattern of covariation between tasks. Across all figures, experimental tasks are presented in the top row, in grey. Factors are shown in the lower row, in colour. The “Intelligence” factor (purple) includes the effect of RPM, FSIQ or both, depending on the variable and group. These models describe the significant contribution of a given factor (i.e., intelligence or other factor) to the variance of any given perceptual task performance. **A.** Generic model. Arrows from the same factor (here, intelligence) pointing towards two tasks (here, 1 and 2) indicate that the correlation between these two tasks can be explained by their common relationship with the factor, represented here as intelligence. In the example presented, the intelligence factor does not fully explain the variance of tasks 1 and 2, and a residual covariation attributed to “another factor” (orange), not dependent on intelligence, explains this residual correlation. **B.** (TD controls) and **C.** (Autistic individuals). Models that fit the observed patterns of covariation in this study for each group separately (statistics available in Table 2 and 4). The factors not dependent on intelligence, that contribute to residual covariations include: the “Unimodal Auditory Aptitude” factor (green), the “Unimodal Visual Aptitude” factor (blue) and the “Plurimodal Perceptual Aptitude” factor (orange). The “Unimodal Auditory Aptitude” factor is a common factor found in both autistic individuals and in the general TD population and explains the relationship between levels of processing within a single perceptual modality. The “Unimodal Visual Aptitude” factor is an analogue to the “Unimodal Auditory Aptitude” factor, but within the visual modality. This factor reaches significance only in the autistics group in the current study. The “Plurimodal Perceptual Aptitude” factor is different from the unimodal aptitude factors and is present only in autistic individuals. This factor is the main finding of the current study and is given the abbreviated “*p*-factor” label in the discussion. Full Lines: *p*<0.05; Dotted lines: *p*<0.1.

The main purpose of this study was therefore to determine how perceptual performances are associated within or across processing levels (low- and mid-level) and/or modalities (visual and auditory) in autism. We sought to examine the effect of intelligence on perceptual performances, and whether patterns of covariation differ between autistic and non-autistic individuals. Based on studies of intelligence, we expect that covariation between perceptual abilities are explained by general intelligence in typically developing individuals. In contrast, in autistic individuals, we expect to find residual covariation between perceptual abilities that is explained by another factor besides general intelligence. This residual covariation would be indicative of a hidden factor, which exerts a common influence on perceptual tasks, irrespective of intelligence (Figure 1a).

**Figure 2:**
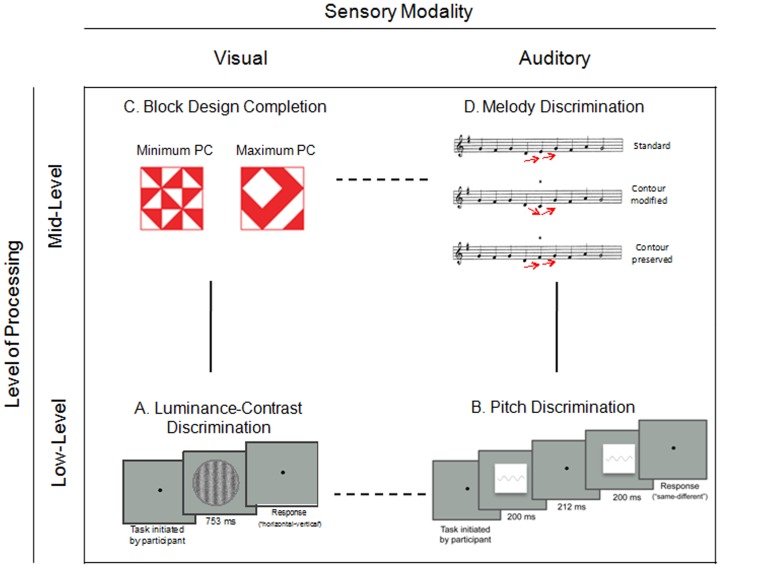
Schematic representation of the study's factorial design and presentation of experimental stimuli and tasks. The four experimental tasks are presented in each quadrant. Each task is characterized by a sensory modality (visual or auditory) and by a level of cortical processing engaged during task completion (low- or mid-level). **A.** Luminance-contrast (LC) discrimination: gratings were presented for 753 ms each and separated by an inter-stimulus interval of 271 ms, during which a noise mask was presented to minimize spatial after effects. **B.** Pitch discrimination: pure tones were presented for 200 ms each and separated by an inter-stimulus interval of 212 ms. **C.** Block design completion: examples of minimum and maximum perceptual cohesiveness (PC) models. **D.** Melody discrimination: examples of a standard melody compared to contour modified and contour preserved conditions. Red arrows represent contour direction. Lines represent relationships of interest in the current study. Full lines: unimodal relationships, between levels of processing; Dotted lines: plurimodal relationships, within levels of processing.

We chose a luminance-contrast discrimination task [39] and a modified block design task [40] to examine low- and mid-level visual processes, respectively. For auditory perception, we used a low-level pitch discrimination task and a mid-level melody discrimination task [41]. Tasks were chosen on the basis of evidence suggesting that they are able to detect high performances associated with autism. These tasks were selected to examine both the relationship between modalities and between different levels of processing. Figure 2 provides a schematic representation of the study's factorial design.

## Methods

### Participants

The target clinical population was comprised of adolescents and adults on the Autism Spectrum (AS). Forty-six autistic individuals and 46 typically developing (TD) participants completed the study. Most autistic individuals had delayed or abnormal language development, because this particular subgroup has been shown to have superior perceptual performance more consistently than AS individuals without developmental language abnormalities [10]. All but three autistic participants presented either a delay in speech onset (30/46) defined according to the ADI-R (first word onset after 24 months or first phrase onset after 33 months) or a score >1 on any of the following ADI items: immediate echolalia, stereotyped speech/delayed echolalia or pronoun reversal suggesting atypical language development (11/46). Standardized information on language development was not available for two participants without language delay or atypicalities in language development. These individuals were diagnosed with autism on the basis of expert clinical judgment and DSM-IV criteria only. Participants were recruited from the «Centre d'Excellence des Troubles Envahissants du Développement de l'Université de Montréal» (CETEDUM) database. No Research Resource Identifier (RRID) can be provided for this population database because it is not publically available. Nonetheless, additional information regarding population characteristics can be requested within the limits of confidentiality. Forty-three autistic participants were diagnosed with the Autism Diagnostic Interview (ADI-R) [42] and/or the Autism Diagnostic Observation Schedule (ADOS-G) [43] (ADI only: three; ADOS only: two; ADI+ADOS: 38). Trained clinical professionals working at the specialized clinic at the Rivière-des-Prairies Hospital carried out both diagnostic tests. Three autistic participants were diagnosed based on DSM-IV criteria and expert (LM) clinical judgment. Neither TD participants nor their first-degree relatives had any history of AS, or other neurodevelopmental or neurological conditions. All participants completed the Autism Quotient (AQ) questionnaire [44]. One control participant reached a score of 30, which is above the recommended cut-off score of 26 [44]. This participant was excluded from the analysis of auditory tasks due to formal musical training; however, the participant was included in the analysis of visual tasks because his performance was comparable to that of other TD controls. All of the participants had a full scale IQ score above the range of intellectual disability (FSIQ greater than 70). Participants who could not complete practice trials (conducted a maximum of 3 times) and those who scored minimally in the first test items or did not complete enough trials to obtain a valid threshold measure, were excluded. The number of excluded participants by analysis is available as supporting information (Table S1). In addition, eight controls and three autistic participants with more than five years of formal music education, as measured with an in-house 20-item questionnaire, were excluded from the auditory task analyses [45], [46]. Table 1 shows the descriptive statistics for age, AQ, intelligence, and baseline motor speed. Participants and their caregiver, for those under the legal age, gave informed, written consent. The research ethics committee (CER) of the Riviere-des-Prairies Hospital approved the study.

**Table 1:** Descriptive statistics for all participants including age and Wechsler's Intelligence Scale IQ (FSIQ, VIQ, NVIQ) and Raven Progressive Matrices (RPM) scores: mean (standard deviation); range.

	TD Controls	Autistic Individuals	Statistics	*p*
n (males: females)	46 (38M: 8F)	46 (38M: 8F)	-	-
Age in years	20.20 (3.74); 18–28	20.57 (5.83); 14–36	t(76.669) = −.362	.718
Autism Quotient	13.74 (4.55); 7–30	25.93 (8.22); 9–45	t(70.177) = −8.802	<.001***
Wechsler FSIQ^a^	108.65 (12.59); 80–131	94.70 (15.41); 71–130	t(90) = 4.757	<.001***
VIQ	108.89 (12.23); 78–128	92.86 (19.64); 47–128	t(67.765) = 4.534	<.001***
NVIQ	107.33 (13.64); 79–133	99.70 (13.54); 71–131	t(88) = 2.659	.009**
RPM^b^	67.13 (23.00); 23–99	71.30 (22.10); 10–99	U = 927	.306
Motor speed in seconds	12.63 (3.63); 8.28–22.54	13.40 (3.42); 7.60–21.69	t(82) = −.990	.325

a Standard scores on the Wechsler's Intelligence Scales;

b Percentile on the Raven Progressive Matrices; TD: Typically Developing, FSIQ: Full Scale IQ (n total = 92), VIQ: Verbal IQ (n total = 87), NVIQ: Non-verbal IQ (n total = 90), RPM: Raven Progressive Matrices (n total = 92); Significance levels:

*p<0.05,

**p<0.01,

***p<0.005.

### Stimuli and Procedure

All participants underwent preliminary visual and auditory acuity testing with standard tests [Runge test and Snellen chart for vision and a pure-tone audiogram (250–8000 Hz) for audition] and had normal to correct-to-normal vision; none of the participants had hearing aids or hearing loss. Testing was conducted in the auditory testing room of the Perceptual Neurosciences Laboratory (PNLab) for Autism and Development, located at Rivière-des-Prairies Hospital. This room is designed to minimize external noise and light sources. Auditory and visual stimuli were produced and presented via the DataPixx graphics and data acquisition toolbox and run on an Apple Macintosh G4 platform with an 18-inch Viewsonic E90FB .25 CRT (1280×1024 pixels) monitor refreshed at a rate of 75 Hz. The background of the display was kept as a grey colour (x = 0.2783|y = 0.321) with an average intensity of 40 cd/m^2^, with a minimum (L_min_) luminance value of 0.50 cd/m^2^ and a maximum (L_max_) luminance value of 89.50 cd/m^2^. Auditory stimuli were presented to both ears simultaneously with Sennheiser HD280 earphones at an intensity of 65 db SPL. The stability of parameters defining the auditory and visual stimuli was assessed with a Quest 1100 sonometer and Minolta CS-100 colorimeter, respectively. All participants underwent preliminary visual and auditory acuity testing with standard tests [Runge test and Snellen chart for vision and a pure-tone audiogram (250–8000 Hz) for audition] and had normal to correct-to-normal vision; none of the participants had hearing aids or hearing loss. The auditory and visual tasks were carried out in a semi-randomized order to counterbalance for crossover, learning, and/or fatigue effects. Tasks involving the same modality were never presented in succession.

#### Low-level Visual Task

Luminance-contrast (LC) discrimination was examined with a task from a study by Bertone et al. (2005) [39]. In this task, participants are asked to fixate a dot on a screen and identify the spatial orientation of sine-wave static gratings by pressing the “right” or “up” arrow of a standard computer keyboard to indicate horizontal or vertical orientation, respectively. Gratings are a regularly spaced collection of identical, parallel, elongated elements. A sine-wave grating is a repeated number of fuzzy dark and light bars, or cycles defined by its level of luminance. An example of a vertically oriented sine-wave grating is shown in Figure 2a. Gratings were presented alone on the screen and the participant initiated each subsequent trial by pressing the spacebar of the keyboard; no time limit was imposed. The task started with five practice trials in which the participant needed to reach a minimum score of 80% correct before continuing to the experimental task. The task used a constant stimuli procedure to determine the identification threshold. Stimuli were presented 10 times for each level of luminance modulation (10%, 5%, 3.5%, 2%, 1.25%, and 0.625%) and for each orientation for a total of 120 trials. Spatial frequency was kept constant at 1 cpd. A Weibull psychometric function [47] was then applied to the set of responses to determine the discrimination threshold for a performance level of 75% correct. This measure of LC discrimination threshold served as the variable of interest (the lower the threshold score, the better the performance). Given that values were very small, LC discrimination threshold scores multiplied by 100 were used in the analyses. This linear transformation had no effect on statistical significance.

#### Low-level Auditory Task

Pitch discrimination was used to investigate low-level auditory processing. Participants listened to a standard pure-tone of fixed value (500, 1000, or 1500 Hz), followed or preceded by a comparison pure-tone, and were asked to indicate whether the two stimuli perceived were the same or different (Figure 2b). Participants held a VPixx response box in each hand and pressed the top button of the box in the left hand if they thought that the pitch was the same and the top button of the box in the right hand if they thought that it was different. Subsequent trials were initiated 750 ms after the participant pressed the bottom button with either hand; no time limit was imposed. Thresholds for the three standard stimuli were obtained within a single adaptive staircase procedure, Harvey's ML-PEST [48]. Thresholds were measured 3 times and averaged to obtain an accurate threshold estimation for each standard stimulus. Before the experimental trials, each participant completed 10 practice trials and had to reach a minimum score of 80% correct responses before continuing the experimental task. The average pitch discrimination threshold served as the variable of interest (the lower the threshold score, the better the performance). The discrimination threshold is expressed as a Weber fraction (*w*): the fractional change in frequency required to discriminate each standard condition (Δ f/f), where Δ f is the minimum difference in frequency required to discriminate accurately between standard and comparison stimuli, and f is the reference standard frequency.

#### Mid-Level Visual Task

Hierarchical local-global processing of visual information was investigated with a modified version of the Block Design subtest of Wechsler's Intelligence Scale from Caron et al. (2006) [21]. This block design task incorporates a large range of difficulty and target figures that vary in their level of perceptual cohesiveness (PC). The PC level is manipulated by changing the number of opposite-coloured edges, or *edge cues*. The higher the number of edge cues, the lower the PC and the faster the task is completed. There were three PC levels: minimum, intermediate and maximum (Figure 2c), corresponding to easy, moderate and hard levels of difficulty, respectively. Task difficulty also increased with the number of blocks included in the model (4, 9, or 16). Participants were encouraged to work as quickly and precisely as possible. A time limit of 120, 180, and 240 seconds was established for the 4, 9 and 16 block models, respectively. A measure of baseline motor speed was obtained by administering a control condition consisting of a plain red target figure for each model size. Completion time for construction of the target figure was recorded, in seconds, with a standard stopwatch. Timing began upon presentation of the model and ended once the figure was completed or the time limit was reached. The variable of interest was the average completion time of the most difficult maximum PC condition in the six different trials (the faster completion time, i.e. the lower the score, the better the performance). Performance in the maximum PC condition was chosen as the variable of interest because it detects differences between groups with the highest level of sensitivity.

#### Mid-Level Auditory Task

Hierarchical local-global processing of auditory information was investigated with a melody discrimination task inspired by an earlier study by Peretz (1987) [49]. The melodies were made up of nine notes each lasting 350 ms except for the last note which lasted twice as long (700 ms). In the “contour-modified condition”, a change in the frequency of one note resulted in a change in the interval direction, whereas in the “contour-preserved condition” a change in frequency did not alter the interval direction (Figure 2d). The modified note was either at the beginning, middle or end of the melody, but never the first or last note. There were a total of 48 trials, including 12 identical melodies presented twice, 12 contour-modified melodies and 12 contour-preserved melodies. Participants had to determine whether the two melodies presented were the same or different. Similar to the pitch discrimination task, participants held a VPixx response box in each hand and pressed the top button of the box in the left hand if they thought that the two melodies were the same and top button of the box in the right hand if they thought that they were different. The task started after two successful practice trials. Sensitivity d-prime values were calculated for each condition, which included 12 “different” and 24 “same” trials. The variable of interest was the level of sensitivity to changes in the most difficult contour-preserved condition, regardless of when the note change was made (beginning, middle or end). Performance in this condition was chosen as the variable of interest because it reflects sensitivity to local changes within a non-automatic situation that generally promotes global processing. For consistency with the other measures, mean d′ scores are presented as inverted values, such that the lower the score, the better the performance. All values were converted to positive values by subtracting d′ scores from a constant: inverted melody discrimination score = 4−d′. This linear transformation had no effect on statistical significance.

### Statistical Analyses

Experiments were designed and the results were interpreted to answer four questions, using two regression models. *Model 1:* 1) For each perceptual task, is the association between intelligence and performance the same for autistic and control groups? 2) Does the relationship between intelligence and performance depend on the measure of intelligence used (FSIQ or RPM)? 3) Does controlling for intelligence with FSIQ or RPM affect differences in performance between groups? *Model 2:* 4) Do patterns of covariation in task performances differ between groups?

#### Model 1: Effect of intelligence on performance and between group-differences in performances

Regression analyses conducted separately for each task and each measure of intelligence examined the effect of intelligence on performance and differences between groups in performance, with the following multiple linear regression model:
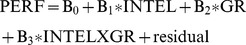
where PERF = task performance, INTEL: intelligence measure, RPM or Wechsler's FSIQ (z-scores), GR: group variable (0 if TD control, 1 if AS)

According to the coding scheme of the independent variables, B_1_ indicates the expected linear increase in mean performance for a one SD increase along the intelligence scale, for the control group. B_3_ indicates the expected between groups difference in linear increase in mean performance for a one SD increase along the intelligence scale. That is, B_1_+B_3_ indicates the expected linear increase in mean performance for a one SD increase along the intelligence scale, for the AS group. B_2_ indicates the expected between groups difference in mean performance, for groups with average intelligence (z-score = 0). When B_3_ is not statistically significant and its estimate near 0, B_2_ indicates the expected between groups difference in mean performance, in this case, constant all along the intelligence scale. When B_3_ is statistically significant or its estimate not close to 0, the expected between groups difference in mean performance vary along the intelligence scale and could be estimated by B_2_+B_3_*INTEL.

#### Model 2: Between group differences in residual covariation in task performance

The following multiple linear regression model was used to analyse how covariation in performances differed between groups, taking into consideration the effect of intelligence (or *residual* covariation):
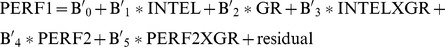
where PERF1 = performance in task 1, PERF2 = performance in task 2 INTEL = intelligence measure (Wechsler's FSIQ or RPM, z-score), GR = group (0 if TD control, 1 if AS).

According to the coding scheme of the independent variables, B_4_ indicates the expected linear increase in mean performance at task 1 for a one unit increase in the performance at task 2 for the control group, while controlling for the effect of intelligence. In other words, B_4_ is the expected residual covariation between tasks for the control group. Similarly, B_5_ indicates the expected difference in residual covariation between groups. Thus, B_4_+B_5_ indicates the expected residual covariation between tasks for the AS group.

Statistical significance was set at *p*<0.05. For each model, assumptions (normality, linearity, homoscedasticity) were checked from residual analysis. Standardised residuals greater than 3 were considered as outliers and were excluded from the analysis. Baseline motor speed was added as an additional covariate if block task performance was part of the regression model (dependent or independent variable).

## Results

### Effect of intelligence on performance

Table 2 shows the main results from model 1. There was a statistically significant FSIQ X Group interaction for pitch and block, but not for the LC and melody discrimination tasks. However, when we controlled for intelligence with RPM, we found a RPM X Group interaction for LC and melody discrimination, but not for the pitch and block tasks. This finding provides a clear yes-answer to our first question, demonstrating that the effect of intelligence on performance is not the same for autistic and controls individuals. Consequently, between group difference in performance vary along the intelligence scale (graphs illustrating these differences are available as Figure S1).

**Table 2:** Model 1 (Effect of intelligence on performance and between group differences in performances) main results: a. Wechsler's Full Scale IQ (FSIQ) or b. Raven Progressive Matrices (RPM).

a.	FSIQ X	FSIQ Simple Effects				Group Effects^a^	
	Group	TD Controls		Autistic Individuals		TD Controls vs AS	
	B_3_	B_1_		B_1_+B_3_		B_2_	
	*p*	estimate	*p*	estimate	*p*	estimate	*p*
LC	.267	−.082	.063	−.145	<.001***	−.050	.396
Pitch	.005**	−.795	<.001***	.006	.976	−1.329	<.001***
Music	.140	−.035	.807	−.311	.011***	−.142	.453
Block^b^	.034 *	−10.233	.001***	−3.348	.149	−15.909	<.001***

b.	RPM X	RPM Simple Effects				Group Effects^a^	
	Group	TD Controls		Autistic Individuals		TD Controls vs AS	
	B_3_	B_1_		B_1_+B_3_		B_2_	
	*p*	estimate	*p*	estimate	*p*	estimate	*p*
LC	.007**	−.045	.329	−.223	<.001***	.278	<.001***
Pitch	.270	−.715	.005**	−.310	.253	−.950	.012*
Music	.020*	−.055	.720	−.551	<.001***	.402	.046*
Block^b^	.760	−6.106	.013*	−4.995	.075	−5.444	.167

For a graphic representation, see Figure S1.

a AS: Autism Spectrum (Autistic) Individuals; TD: Typically Developing; B: Unstandardized regression coefficient. Negative B values indicate that autistic individuals perform better (i.e. lower score) than controls, and vice versa for positive B values;

b Baseline motor speed is added to the model; Significance levels:

*p<.05,

**p<.01,

***p<.005.

Qualitative inspection of simple effects (Table 2) revealed that for TD controls, the association between intelligence and tasks (LC, pitch, and block) was stronger when FSIQ was used as an intelligence measure than when RPM was used. However, for autistic individuals, performance in tasks (LC and melody) was more strongly associated with RPM than with FSIQ. These observations provide a clear yes-answer to our second question, showing that the effect of intelligence on performance differs according to the measure of intelligence used.

It has been proposed that RPM is a better measure of general intelligence than FSIQ in autism; therefore, we carried out a complementary stepwise regression analysis to test the effect of FSIQ over that of RPM among controls, and the effect of RPM over that of FSIQ among autistic individuals. We began the analyses by including the least accurate intelligence measure in the model, and then added the most accurate measure. In controls, FSIQ contributed significantly to the regression model, in addition to the contribution made by RPM, for LC (*p* = .077), pitch (*p* = .005), and block (*p* = .001) tasks. However, in autistic individuals, RPM contributed significantly to the regression model, in addition to the contribution made by FSIQ, for LC (*p* = .002) and melody (*p* = .003) tasks. This finding will be considered during the interpretation of results of residual covariation, because inaccurate measures of intelligence can produce false positive conclusions.

### Between group differences in task performance

Table 3 shows the expected group means, according to model 1, at average intelligence and at one standard deviation above average intelligence. When we controlled for intelligence with FSIQ, autistic individuals performed better in pitch discrimination and block tasks than controls, although there were no significant differences in performance in LC and melody tasks between groups. When we controlled for intelligence with RPM, autistic individuals performed better in the pitch discrimination task, whereas controls performed better in LC and melody discrimination tasks. Performance in the block task was not statistically different between groups. These findings provide a yes answer to our third question, because they show that between group differences in performance depend on the measure used to control for general intelligence (FSIQ or RPM). Interestingly, the difference in performance between groups with average intelligence was larger than between groups with an intelligence level one standard deviation above average, regardless of the measure of intelligence used.

**Table 3:** Expected mean performance according to Model 1 at average intelligence and one SD above average intelligence a. Wechsler's Full Scale IQ (FSIQ) or b. Raven Progressive Matrices (RPM).

a.	Expected Means at FSIQ = 0 SD					Expected Means at FSIQ = +1 SD				
	TD Controls		Autistic Individuals			TD Controls		Autistic Individuals		
	Mean	SE	Mean	SE	*p*	Mean	SE	Mean	SE	*p*
LC	0.832	0.044	0.882	0.039	.396	0.750	0.040	0.737	0.060	.859
Pitch	3.022	0.202	1.692	0.183	<.001***	2.227	0.201	1.698	0.293	.142
Music	2.719	0.140	2.577	0.125	.453	2.684	0.142	2.266	0.201	.093
Block^a^	51.707	2.552	35.798	2.106	<.001***	41.474	2.323	32.450	3.416	.024*

b.	Expected Means at RPM = 0 SD					Expected Means at RPM = +1 SD				
	TD Controls		Autistic Individuals			TD Controls		Autistic Individuals		
	Mean	SE	Mean	SE	*p*	Mean	SE	Mean	SE	*p*
LC	0.809	0.042	1.087	0.051	<.001***	0.765	0.039	0.864	0.036	.066
Pitch	2.920	0.208	1.970	0.303	.012*	2.204	0.231	1.660	0.185	.071
Music	2.783	0.125	3.185	0.154	.046*	2.729	0.139	2.634	0.106	.590
Block^a^	46.147	2.281	40.703	3.145	.167	40.040	2.023	35.707	1.803	.116

a Baseline motor speed is added to the model. For FSIQ: predicted mean for motor speed = 12.998, For RPM: predicted mean for motor speed = 12.875; TD: Typically Developing; SE: Standard Error; Significance levels:

*p<.05,

**p<.01,

***p<.005.

### Between group difference in residual co-variation in task performances

Table 4 shows the main results from model 2. In line with the yes answer to our first question (Is the association between intelligence and performance the same for autistic and control groups?), intelligence X group interaction was included in the 16 multiple linear regression models.

**Table 4:** Model 2 (Between group differences in residual covariation) main results: a. Wechsler's Full Scale IQ (FSIQ), or b. Raven Progressive Matrices (RPM).

a.		Covariation	Covariation by Group			
Independent Variable →		X Group	TD Controls		Autistic Individuals	
Dependent Variable		B_5_	B_4_		B_4_+B_5_	
		*p*	estimate	*p*	estimate	*p*
Low-Level Tasks	Pitch→LC	.011*	−.002	.951	.134	.003***
	LC→Pitch	.179	.042	.964	1.691	.032*
Mid-Level Tasks^a^	Music→Block	.291	4.253	.220	−.438	.876
	Block→Music	.583	.006	.417	−.003	.841
Visual Modality^a^	LC→Block	.921	10.494	.386	9.076	.240
	Block→LC	.025*	.001	.558	.012	.008**
Auditory Modality	Pitch→Music	.452	.263	.005**	.392	.009**
	Music→Pitch	.111	1.003	<.001***	.441	.058

b.		Covariation	Covariation by Group			
Independent Variable →		X Group	TD Controls		Autistic Individuals	
Dependent Variable		B_5_	B_4_		B_4_+B_5_	
		*p*	estimate	*p*	estimate	*p*
Low-Level Tasks	Pitch→LC	.086	.019	.484	.113	.086
	LC→Pitch	.740	1.010	.273	1.423	.094
Mid-Level Tasks^a^	Music→Block	.312	3.524	.308	−.939	.737
	Block→Music	.815	.002	.819	−.002	.893
Visual Modality^a^	LC→Block	.190	24.509	.022*	8.261	.236
	Block→LC	.058	.002	.310	.010	.019*
Auditory Modality	Pitch→Music	.668	.211	.010**	.281	.077
	Music→Pitch	.092	.948	.002***	.296	.240

a Motor speed was also statistically controlled for; TD: Typically Developing, LC: Luminance-Contrast Discrimination Task; SE: Standard Error, B: Unstandardized regression coefficient; Significance levels:

*p<.05,

**p<.01,

***p<.005.

#### Plurimodal co-variation

The upper parts of Table 4 (a and b) show the residual covariation of performances between low- and mid-level tasks. When intelligence was measured by FSIQ (Table 4a), we found significant residual covariation for low-level tasks in autistic individuals. This residual covariation was significantly different from that of the TD control group. However, there was only a trend in covariation for low-level tasks in autistic individuals (*p*<0.10) when intelligence was measured by RPM (Table 4b).

#### Unimodal co-variation

The lower parts of Table 4 (a and b) show results of the residual covariation of performances between visual tasks and auditory tasks. There was significant residual covariation for auditory tasks in both groups when intelligence was measured by FSIQ (4a). In addition, there was significant residual covariation for visual tasks in the autistic group. This residual covariation was significantly different from that of the TD control group. When intelligence was measured by RPM (4b), we found significant residual covariation for auditory tasks in the control group and significant residual covariation for visual tasks in both groups. We found no significant differences between groups, although trends (*p*<0.10) were observed. These findings provide a yes answer to our fourth, and main question, showing a different pattern of residual covariation in task performance between groups.

## Discussion

### Summary of findings

In this study, we report the first systematic assessment of the association of autistic perceptual performance across processing levels (low- vs. mid-) and modalities (auditory vs. visual), and we examine the role of intelligence in this covariation. Our main finding is that perceptual performances in auditory and visual tasks are associated in autistic individuals, and this association cannot be explained by intelligence alone. In the following discussion, we will propose a plausible interpretation of this finding and its relation with intelligence, in the context of the current literature.

#### Comparison of task performances between groups

The autism group outperformed the TD control group in the pitch discrimination (low-level, auditory) and modified block design tasks (mid-level, visual). These findings are consistent with previous studies demonstrating that autistic individuals perform better than non-autistic individuals in similar pitch discrimination tasks, which is now considered to be the most replicated low-level perceptual strength in autism (for a review, see Mottron et al. 2013 [31]). Autistic individuals showed a higher performance in pitch discrimination tasks than non-autistic individuals regardless of whether intelligence was controlled for with Wechsler's FSIQ or RPM. This suggests that such superior low-level auditory performance is not a by-product of a more (RPM) or less (Wechsler's Intelligence Scales) conservative IQ matching strategy. This supports the hypothesis that a fundamental difference in the neural encoding of pitch underlies this autistic perceptual superiority.

In accordance with previous studies, we found that autistic individuals performed better than non-autistic individuals in the modified block design task; however, this was only true when the Wechsler's FSIQ was used as a covariate (see also Stevenson and Gernsbacher, 2013 [50]). Indeed, group differences were no longer observed when we controlled for intelligence with the RPM. This result is consistent with the findings of Dawson et al. (2007) [37]. They demonstrated that autistic individuals who performed on average within the 60^th^ percentile in the block design subtest, versus the 25^th^ percentile for TD controls with the same FSIQ level, had a mean RPM intelligence score also in the 60^th^ percentile. This study demonstrated that particular perceptual performances in autism depend on the matching variable, with some peaks of ability disappearing when groups are matched with IQ measures that do not underestimate intelligence (i.e., RPM). In addition to the mid-level, block design task, a similar matching effect has also been found for other low-level visual tasks, such as inspection time [36].

In contrast, we found no differences between groups for performances in low-level visual and mid-level auditory tasks included in our design. Therefore, our analysis did not replicate previous studies controlling for Wechsler's FSIQ, in which autistic individuals [14] and children at risk of autism [15] were shown to be more sensitive to luminance-defined information than control individuals. Moreover, we found that the TD control group performed better in this task than the autistic group when we controlled for intelligence with RPM. In addition, our results do not replicate previous findings that autism is associated with a local processing advantage for melody processing, because TD controls of average RPM intelligence were more sensitive than autistic individuals to local changes in simple melodies. This discrepancy may be due to differences in task sensitivity between studies. Indeed, Bouvet et al. (2014) [24] showed that autistic individuals were more adept at detecting local elements within musical stimuli than RPM and Wechsler's performance IQ-matched controls; however, global to local interference was poorer in autistic individuals than in controls.

Overall, these findings suggest that although high performance in perceptual tasks is a defining indicator of atypical perception in autism, perceptual particularities cannot be simply interpreted as “stronger than typical” perception. For instance, autism-associated proficiency in the block design test results from autonomy from the top down influence of perceptual cohesiveness, which may or not result in exceptional performance according to the difficulty of the task [21].

### Explaining the residual covariation between tasks beyond the g-factor: the *p*-factor

Figure 1 shows a plausible model that may account for the different pattern of covariation between tasks, and the relationship between tasks and intelligence observed among autistic and non-autistic groups. As indicated in the theoretical model accounting for our results, a significant part of the covariance in task performance could not be explained by intelligence both in the autistic and the TD control group, although different factors may account for this trend in autistic and non-autistic individuals (Table 4). Figure 1b and c shows a plausible model that may account for the different pattern of covariation between tasks, and the relationship between tasks and intelligence observed among autistic and non-autistic groups. For non-autistic participants, all observed covariation could either be explained by a common relationship with intelligence, or with a unimodal, auditory aptitude factor. A residual covariation between visual tasks is found in non-autistics when statistical control is made with RPM, but it vanished when the statistical control is made with FSIQ. Since the FSIQ is considered a more accurate measure than RPM in this group, the unimodal visual aptitude factor is not included in the model. However, in the autistic group, a different pattern of covariation emerges in which covariation across visual tasks and between plurimodal tasks requires additional, explanatory factors.

General intelligence (i.e., the *g*-factor) accounts for around one fifth of the variance in perceptual tasks in the typically developing population. Intelligence is typically related to performance in perceptual tasks such as inspection time [51], motion perception [52] or pitch discrimination [53] in typically developing individuals. According to the Cattell-Horn-Carroll theory [54], several broad abilities are responsible for the variance that cannot be explained by fluid and crystallized intelligence, or by their combination [55]. The proportion of variance that cannot be explained by intelligence increases with IQ, and has been attributed to the differentiation of specific abilities [55]. This explanation may also account for covariation in the autistic group that cannot be explained by the *g*-factor. Current data suggest *the differentiation of perception at large*, in addition to covariation explained by the broad, modality-specific abilities in typical individuals. Accordingly, covariation of performance in low-level tasks involving *two different modalities* (thereby involving anatomically independent processing systems), as well as *two different levels of perceptual integration* (low- and mid-level), indicates that several perceptual abilities may be influenced by a specific plurimodal perceptual aptitude factor in autism. We propose to label this factor the *p*-factor, to distinguish it from the *g*-factor. Such an altered perceptual factor in autism is likely to underlie and generate cascade effects on several cognitive and adaptive mechanisms [56], [57], including, but not limited to, various perceptual abilities across modalities.

Reports of these alterations suggest a model in which several genetic mutations promote the construction of local neural networks [58], which would plausibly have a greater effect on low-level coding mechanisms than subsequent, more complex stages of processing. According to several, recent systematic analyses and reviews, most mutations involved in autism converge toward enhanced plasticity mechanisms [59]. One hypothesis therefore is that the *p*-factor represents the interface between the final common genetic pathway of mutation involved in autism, and neurocognitive cascade effects. Both early visual and auditory sensory systems are selectively responsive to frequency-defined perceptual attributes (spectral or spatial frequency); therefore, we propose that atypical, probably overstimulated, tuning of frequency-selective mechanisms is a type of local alteration common to both perceptual modalities in autism. Frequency-selective mechanisms are modulated by the balance of excitatory/inhibitory activity which encodes elementary information [60]. Both animal and human studies have shown that GABA mediates this balance in both visual and auditory modalities [61]–[63]. The implication of altered lateral GABAergic inhibition in perceptual anomalies in autism is consistent with behavioural, physiological and genetic demonstrations of altered lateral inhibition within early visual areas in autism [56], [64], [65]. There is also evidence that high concentrations of GABA in humans are related to enhanced line orientation [61] and tactile discrimination thresholds [66], supporting the hypothesis that GABAergic mechanisms play an important role in cross-modal alteration of perception in autism.

## Limitations and Conclusions

Our findings may have been influenced by the choice of task. Indeed, the identity of the *p*-factor that is responsible for perceptual covariation is unknown, and some tasks may be more dependent than others on this mechanism. This is the first study of its kind; therefore, the analyses were mostly exploratory and not corrected for multiple analyses. Statistical adjustments (Bonferonni type) became overly conservative when analyses involved correlated independent variables, or when analyses were repeated on multiple correlated dependent variables. Moreover, our analytic strategy was limited by samples size and the number of tasks. We recommend that large scale studies should be carried out in the future. Such studies should include several tasks within and between levels and modalities and use statistical methods such as factor analysis and structural equation modelling to identify the precise components of the perceptual *p*-factor. A similar study involving tasks associated with exceptional performance in autism (e.g. for mid-level auditory level [24] and for low-level visual level [67]) may also unravel strong effects.

The results of multiple linear regression analyses should be considered as underestimates of the link between observable performances and the unobservable *p*-factor. Furthermore, previous studies have assumed equal regression slopes when applying standard methods to control for intelligence (e.g., matching participants within groups). However, as demonstrated in this study, unequal regression slopes can indicate varying group differences along the intelligence scale and/or inaccuracy of the measure of intelligence used. This highlights the importance of testing this assumption whenever sample size and available data make it possible, regardless of the method of control (matching or statistical) and the measure of intelligence (e.g., RPM or Wechsler) chosen. Only then can we make accurate interpretations about the generalization of findings.

## Supporting Information

Figure S1 **Task performance – Intelligence relationships: groups differences.** Raw performance for each experimental task (y axes) plotted on intelligence level (x axes). Autistic individuals are in green, TD controls are in blue. These graphs represent the statistics found in Table 2. The statistics presented in Table 3 can also be visualized on this figure by looking at differential group performances for intelligence levels at 0SD and +1SD. Note that the graph for the block design task does not exactly illustrate the statistics from Table 2 and 3 since a 2D representation of the data could not include motor speed as a covariate.
(TIF)

Table S1 Number of participants excluded a. in each task, because of failure to complete task. Numbers in Table S1.a. also include the 3 autistic and 8 control participants with musical experience who are excluded solely from auditory task (i.e., Pitch and Music), and b. within regression analysis because of residuals >3 standard deviations.
(DOC)
