# Supplementary figures and images for: Autism-Specific Covariation in Perceptual Performances: “g” or “p” Factor?
Source: PLoS One. 2014 Aug 12;9(8):e103781. doi: 10.1371/journal.pone.0103781 (PMC4130524; doi:10.1371/journal.pone.0103781)

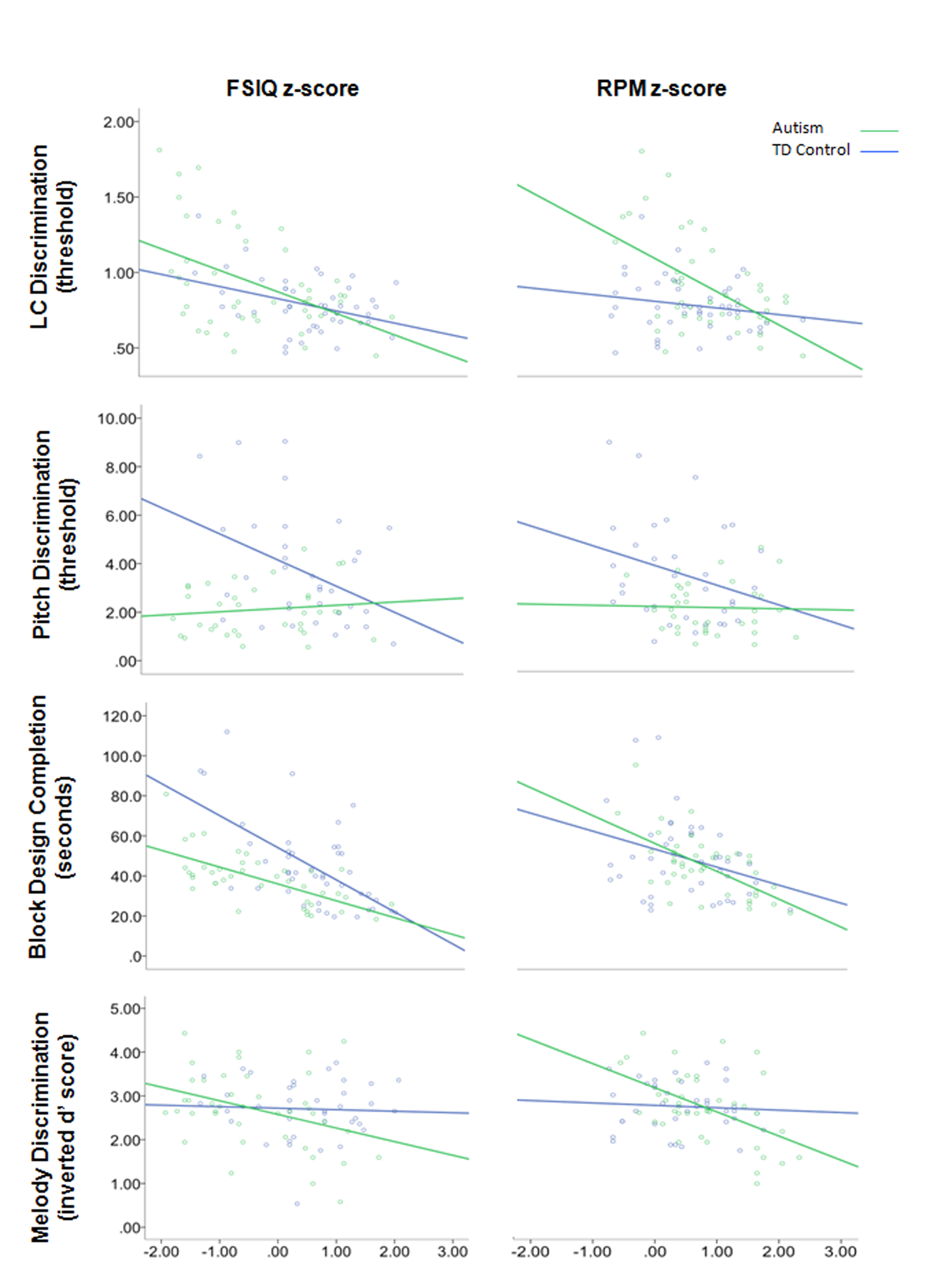

Supplement: Figure S1 — Task performance – Intelligence relationships: groups differences. Raw performance for each experimental task (y axes) plotted on intelligence level (x axes). Autistic individuals are in green, TD controls are in blue. These graphs represent the statistics found in Table 2. The statistics presented in Table 3 can also be visualized on this figure by looking at differential group performances for intelligence levels at 0SD and +1SD. Note that the graph for the block design task does not exactly illustrate the statistics from Table 2 and 3 since a 2D representation of the data could not include motor speed as a covariate. (TIF) [file pone.0103781.s001.tif]
