# Supplementary material for: Autism-Specific Covariation in Perceptual Performances: “g” or “p” Factor?
Source: PLoS One. 2014 Aug 12;9(8):e103781. doi: 10.1371/journal.pone.0103781 (PMC4130524; doi:10.1371/journal.pone.0103781)
Supplement: Table S1 — Number of participants excluded a. in each task, because of failure to complete task. Numbers in Table S1.a. also include the 3 autistic and 8 control participants with musical experience who are excluded solely from auditory task (i.e., Pitch and Music), and b. within regression analysis because of residuals >3 standard deviations. (DOC) [file pone.0103781.s002.doc]

| **Table S1.** Number of participants excluded a. in each task, because of failure to complete task. Numbers in Table S1.a. also include the 3 autistic and 8 control participants with musical experience who are excluded solely from auditory task (i.e., Pitch and Music), and b. within regression analysis because of residuals >3 standard deviations. | | | | | | |
| --- | --- | --- | --- | --- | --- | --- |
| a. |  | TD Controls | |  | Autistic Individuals | |
|  |  | Number of Subjects Excluded | Final Group Sample Size |  | Number of Subjects Excluded | Final Group Sample Size |
|  | Pitch | 13 | **33** |  | 12 | **34** |
|  | LC | 0 | **46** |  | 2 | **44** |
|  | Music | 9 | **37** |  | 3 | **43** |
|  | Block | 2 | **44** |  | 6 | **40** |
|  |  |  |  |  |  |  |
| b. |  | Controlling for FSIQ | |  | Controlling for FSIQ | |
| Independent Variable 🡪 | | Number of | Final Full |  | Number of Subjects | Final Full |
| Dependent Variable | | Subjects Excluded | Sample Size |  | Excluded | Sample Size |
| Low-Level | Pitch🡪LC | 0 | **65** |  | 0 | **65** |
| Tasks | LC🡪Pitch | 1 TDC | **64** |  | 1 TDC | **64** |
| Mid-Level | Music🡪Block | 0 | **71** |  | 1 TDC | **70** |
| Tasks | Block 🡪Music | 0 | **71** |  | 0 | **71** |
| Visual | LC🡪Block | 0 | **82** |  | 2 TDC | **80** |
| Modality | Block 🡪LC | 0 | **82** |  | 0 | **82** |
| Auditory | Pitch🡪Music | 0 | **64** |  | 1 AS | **63** |
| Modality | Music🡪Pitch | 0 | **64** |  | 0 | **64** |
| TDC: TD Control; AS: Autism Spectrum (Autistic) Individual; LC: Luminance-Contrast Discrimination Task. | | | | | | |
